# Effects of ganglioside G(M1) and erythropoietin on spinal cord lesions in rats: functional and histological evaluations

**DOI:** 10.6061/clinics/2016(06)11

**Published:** 2016-06

**Authors:** Raphael Martus Marcon, Alexandre Fogaça Cristante, Tarcísio Eloy Pessoa de Barros Filho, Ricardo Ferreira, Gustavo Bispo dos Santos

**Affiliations:** Instituto de Ortopedia e Traumatologia, Hospital das Clínicas da Faculdade de Medicina da Universidade de São Paulo (IOT-HCFMUSP), Divisão de Cirurgia de Coluna Vertebral, Laboratório de Investigação Médica (LIM 41), São Paulo/SP, Brazil

**Keywords:** Erythropoietin, Gangliosides, Ganglioside G(M1), Spinal cord compression, Spinal cord, Rats, Wistar

## Abstract

**OBJECTIVE:**

To evaluate the functional and histological effects of ganglioside G(M1) and erythropoietin after experimental spinal cord contusion injury.

**METHODS:**

Fifty male Wistar rats underwent experimental spinal cord lesioning using an NYU-Impactor device and were randomly divided into the following groups, which received treatment intraperitoneally. The G(M1) group received ganglioside G(M1) (30 mg/kg); the erythropoietin group received erythropoietin (1000 IU/kg); the combined group received both drugs; and the saline group received saline (0.9%) as a control. A fifth group was the laminectomy group, in which the animals were subjected to laminectomy alone, without spinal lesioning or treatment. The animals were evaluated according to the Basso, Beattie and Bresnahan (BBB) scale, motor evoked potential recordings and, after euthanasia, histological analysis of spinal cord tissue.

**RESULTS:**

The erythropoietin group had higher BBB scores than the G(M1) group. The combined group had the highest BBB scores, and the saline group had the lowest BBB scores. No significant difference in latency was observed between the three groups that underwent spinal cord lesioning and intervention. However, the combined group showed a significantly higher signal amplitude than the other treatment groups or the saline group (*p*<0.01). Histological tissue analysis showed no significant difference between the groups. Axonal index was significantly enhanced in the combined group than any other intervention (*p*<0.01).

**CONCLUSION:**

G(M1) and erythropoietin exert therapeutic effects on axonal regeneration and electrophysiological and motor functions in rats subjected to experimental spinal cord lesioning and administering these two substances in combination potentiates their effects.

## INTRODUCTION

Ganglioside G(M1) is a therapeutic option for the treatment of lesions of the central nervous system (CNS) 1. The various properties attributed to G(M1) include the reduction of neural edema by increasing the activities of sodium, potassium and magnesium pumps; the homeostasis of neural cells by reestablishing membrane equilibrium 2; specifically increasing the levels of endogenous neurotrophic factors, thus reducing the destruction of neurons following trauma; inducing the plasticity mechanisms of injured spinal circuits; and promoting the recovery of functional connections 3. Research involving G(M1) in humans has shown that this treatment improved locomotor functions in victims of spinomedullary trauma 4, but the interpretation of these results is complicated because methylprednisolone had been administered before G(M1) treatment 5.

Erythropoietin is a glycoprotein produced in the kidneys of adults. This substance can mediate cytoprotection in various tissues, including nervous tissue. Inhibition of apoptosis, reduction of the inflammatory process, restoration of vascular integrity and regeneration of neurons are the primary activities attributed to this glycoprotein 6,7. Erythropoietin stands out among the substances used in neuroprotective therapy. *In vivo*, its neuroprotective properties have proven effective in studies using animal models of ischemia, closed trauma, epilepsy and spinal lesioning. The cellular and molecular mechanisms of this neuroprotective agent remain uncertain 8. Erythropoietin also acts on microglia, which are hematopoietic in origin, exhibit high cell plasticity and play important roles in the immune system and in the repair of the CNS 9.

This study was motivated by the possibility of the synergetic use of G(M1) and erythropoietin as an adjuvant treatment of spinal lesions based on a consistent line of evidence from studies of experimental lesions in rats 10-15. The use of these two substances together indicates a possible breakthrough in the quality of neural regeneration, stemming from the principle that minimal anatomical repairs of the spinal cord can result in clinically significant improvements in patients who experience spinal cord lesions. Although the ability to walk may not be restored, axonal regeneration, even if partial, may result in the recovery of functions such as sphincter control, or upper limb function – improvements that can translate to significant increases in the autonomy of patients, who are often young.

## OBJECTIVES

To evaluate the functional and histological effects of treatment with monoganglioside G(M1) and erythropoietin in spinal cord contusion lesions in Wistar rats.

## METHODS

### Design, ethics and animals

The research protocol for this experimental study involving animals was evaluated and approved by the Research Ethics Committee of our institution. The research laboratory strictly adhered to all the international guidelines on handling and pain control related to the care and use of animals in research. Five animals were housed in each cage in the laboratory and the animals were handled and induced to move prior to the experiment so that they could become accustomed to the researchers and to the experimental evaluation of motor function after spinal cord injury. Ad libitum feeding and hydration were maintained throughout the study.

Sixty male Wistar rats, aged 20 to 21 weeks and weighing from 254 to 405 g, were used. All rats were weighed at the beginning and the end of the study. All of the rats had normal coats, normal clinical status and normal movement capability at the beginning of the study. The sample size was based on previous studies using 10 animals per group 12,15. The experimental spinal cord lesion was confirmed using the Basso, Beattie and Bresnahan (BBB) scale, indicating the lack of normal movement of the legs.

The following exclusion criteria were established: death following experimental spinal cord lesioning, macroscopic observation of abnormalities in the area of the spinal cord lesion and autophagy or mutilation of the animals during the observation period.

The rats were randomly divided into five groups of 12 animals per group:

- G(M1) group – rats undergoing experimental spinal cord lesioning and receiving G(M1) ganglioside intraperitoneally (30 mg/kg);
- Erythropoietin group – rats undergoing experimental spinal cord lesioning and receiving erythropoietin intraperitoneally (1000 UI/kg);
- Combined group – rats undergoing experimental spinal cord lesioning and receiving both G(M1) (30 mg/kg) and erythropoietin (1000 UI/kg) intraperitoneally;
- Saline group – rats undergoing experimental spinal cord lesioning and receiving saline (0.9%) intraperitoneally (control group);
- Laminectomy group – rats undergoing laminectomy alone, without spinal cord lesioning and treatment.

### Experimental spinal cord lesioning and experimental interventions with G(M1) and erythropoietin

In this study, all rats, even those not subjected to spinal cord lesioning, underwent laminectomy under a surgical microscope as a standardized procedure 11,14,15. Prior to laminectomy, experimental spinal cord lesioning, motor evoked potential (MEP) recording and euthanasia, the animals were anesthetized via intraperitoneal injection of 10 mg/kg xylazine and 50 mg/kg ketamine. The rats were examined to confirm anesthesia as described elsewhere 14-16. A moderate contusion lesion was produced at T10 using an NYU-Impactor device as previously described 14,15; a 10-g impact rod was released from a standardized height of 12.5 mm to compress the spinal cord of all rats for 15 seconds.

The animals received preventative antibiotic therapy (sodium cefazolin, 20 mg/kg, intraperitoneally) immediately after the lesion and once a day for three days; this treatment was extended to seven days in cases of persistent infection. Signs of blood in the urine were considered to indicate an untreatable urinary tract infection, prompting exclusion from the study and euthanasia to prevent contamination of the other animals.

Following spinal cord lesioning, pain control was achieved by intramuscularly injecting 2 mg/kg meloxicam (once daily for seven days) and tramadol chlorhydrate (5 mg/100 g, once daily for seven days). The medications were administered to the animals in all groups, including the control group, for the same treatment duration.

Due to the loss of the micturition reflex as a result of the spinal cord lesion, the bladder of the animals was manually emptied twice daily until the animals regained bladder function, which usually occurred on the third postoperative day. The animals were observed for signs of infection, dehydration, mutilation and autophagic behavior until the 42^nd^ postoperative day, when they were sacrificed.

The study drugs were administered intraperitoneally at the doses described above, always with the rats under anesthesia and sedated. In the combined group, the injections were applied at different sites in the peritoneum using different syringes.

### Evaluation of locomotor function

The recovery of locomotor function was evaluated for 4 to 5 minutes using the BBB scale on days 2, 7, 14, 21, 28, 35 and 42 after spinal cord lesioning. Each rat was assessed simultaneously by two suitably trained observers who had no knowledge of the source group of the rats and were blinded to their colleague’s evaluations, so as to avoid interfering with each other’s results. When there was disagreement between the evaluations, the lower score was recorded for analysis.

The rats also underwent MEP recordings of the amplitude and latency of the responses in the paws on both sides following transcranial electrical stimulation 13. MEP was performed under anesthesia on the 42^nd^ day after spinal cord lesioning, the day of euthanasia. Muscular responses were recorded by inserting pairs of monopolar needle electrodes (sensor and reference electrodes) at a defined and consistent inter-electrode distance into the proximal and anterior musculature of the right and left hind limbs 13. A monopolar needle electrode was placed in the lumbar region as a ground. Transcranial electrical stimulation was applied by inserting two corkscrew-type electrode needles into the head of the rats along the inter-hemispheric line in the frontal (anode) and occipital (cathode) lobes for simultaneous bilateral stimulation. The device was calibrated in accordance with the standards previously published by our team and the transcranial electrical stimulation consisted of a single stimulus of 0.2 ms in duration.

### Euthanasia

At the end of the experimental period (after 42 days), all the rats were euthanized according to the ethical guidelines for animal experimentation. The euthanasia procedure was performed in three steps: anesthesia (with ketamine and xylazine), cardiac perfusion (for three minutes with a 0.2 M phosphate buffer solution at pH 7.4 and 36°C, followed by a 4% paraformaldehyde buffer solution at pH 7.4, 100 ml/100 g) and painless death (via intravenous injection of 5 ml of potassium chlorate (at 19.1%)).

### Necropsy and histological tissue analysis

After euthanasia, the presence of macroscopic abnormalities, such as autophagic or mutilation lesions, was evaluated in the autopsy. The lungs and the bladder were also evaluated.

The spinal cord was examined and removed at the site of the lesion, from T8 to T12 (approximately 2.5 cm in length). The extracted segment was fixed and prepared for histological analysis as described previously to detect the presence of necrosis, hemorrhage, hyperemia, degeneration and infiltrates 12.

Two representative 2-µm-thick sections from each spinal cord specimen, 1 mm distal and 1 mm proximal to the center of the lesion, were selected, fixed in osmium tetroxide solution (2%) and stained with toluidine blue (1%) for axonal counting 12,15.

Photographs at 40 x magnification were analyzed using SigmaScan Pro 5.0 software. Neurons with a diameter equal to or greater than 15 µm were counted 17. The following formula was used to calculate the regeneration index (RI):

RI = (number of axons in the distal area/number of axons in the proximal area) x 100 18.

A single experienced pathologist who was blinded to the rat allocation performed the analyses.

### Statistical analysis

The primary outcome evaluated in this study was the BBB score on the 42^nd^ postoperative day. Histological evaluation variables and MEP results were considered as secondary outcomes.

Data were expressed as means and standard deviations and were tested for normality using the Kolmogorov-Smirnov test. When the data presented a normal distribution, they were evaluated using parametric tests.

Student’s t test for paired samples was used to compare the right and left sides. One-way analysis of variance (ANOVA) was used to compare the five groups. The chi square test was only used for the axon counting results. Bonferroni correction was used for post hoc multiple comparison testing. The Kruskal-Wallis test was used for the analysis of the BBB scores between groups. For the comparison of the results between weeks in a given group, the Friedman test was used because the data were not normally distributed (*p*<0.05).

We started with a null hypothesis, considering a probability of type I error of 5%. The Statistical Package for the Social Sciences (SPSS), version 19.0 for Windows, was used in the analysis.

## RESULTS

The five groups of rats underwent the planned interventions without technical complications. Two rats from each group were excluded because of autophagic behavior and infection. Weight gain occurred in the G(M1), combined and saline groups (averages of 6.6 g, 5.9 g and 50.3 g, respectively), but slight weight loss occurred in the erythropoietin and laminectomy groups (6.8 g and 23.1 g, respectively). Considering that the initial mean weight of the rats was 346.52 g, the average total weight gain was 6.58 g.

### Functional analysis: Locomotion

**Figure 1:**
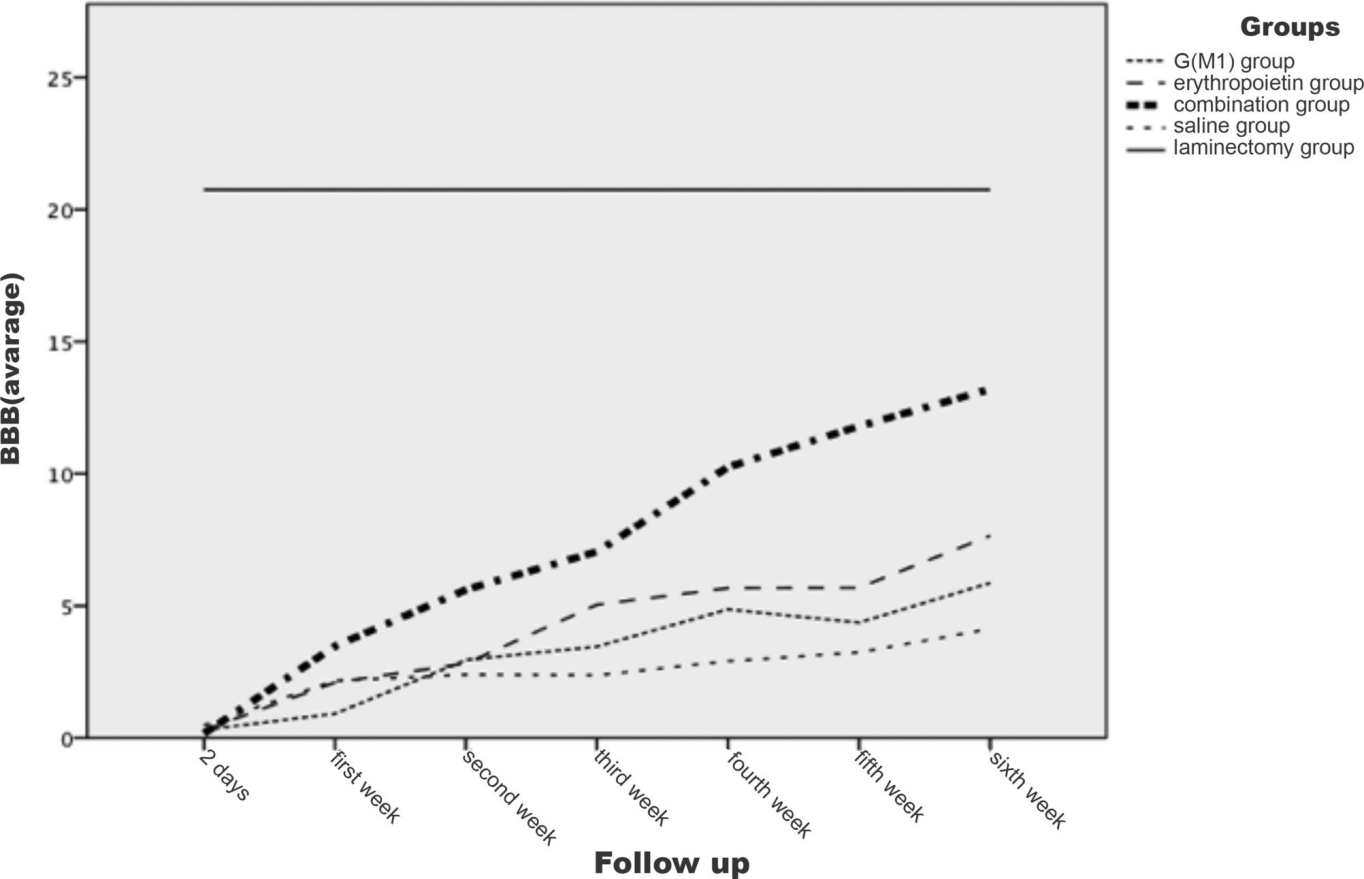
BBB scores over time.

The evaluation of motor function using the BBB scale, conducted at seven different time points, showed no difference between the right and left sides of the rats (*p*>0.1). Thus, for the comparison between groups, the data for the right and left sides were combined. The evolution of the BBB scores is shown in Figure 1. All groups exhibited increasing BBB scores over time and these increases were statistically significant beginning from the second week of analysis (*p*<0.05).

On the first evaluation, conducted two days after the trauma and intervention, no statistically significant difference in locomotion between the groups subjected to trauma was observed (*p*>0.05). Only the laminectomy group (which was not subjected to spinal cord lesioning) showed a difference in locomotion compared to the saline group and the intervention groups (*p*<0.05). Beginning with the second evaluation, performed one week after spinal cord lesioning (or laminectomy), the groups began to show statistically significant differences in locomotion (*p*<0.05). In this second evaluation, the erythropoietin group exhibited significantly higher BBB scores than the G(M1) group (*p*=0.002). Moreover, the combined (G(M1) and erythropoietin) group exhibited significantly higher BBB scores than all other groups subjected to spinal cord lesioning (*p*<0.05). The saline group exhibited the lowest BBB scores at all time points examined (*p*<0.05) and the laminectomy group exhibited higher BBB scores than the other groups at all time points (*p*<0.05).

### Functional evaluation via motor evoked potential recording

The MEP recordings provided data for the signal latency and amplitude on both sides of the examined rats. No statistically significant difference in either latency or amplitude was found between the two sides (*p*>0.05). Thus, comparison between the groups was performed on the pooled data of the right and left sides of the rats in each group. The detailed results are presented in Tables 1 and 2.

**Figure 2:**
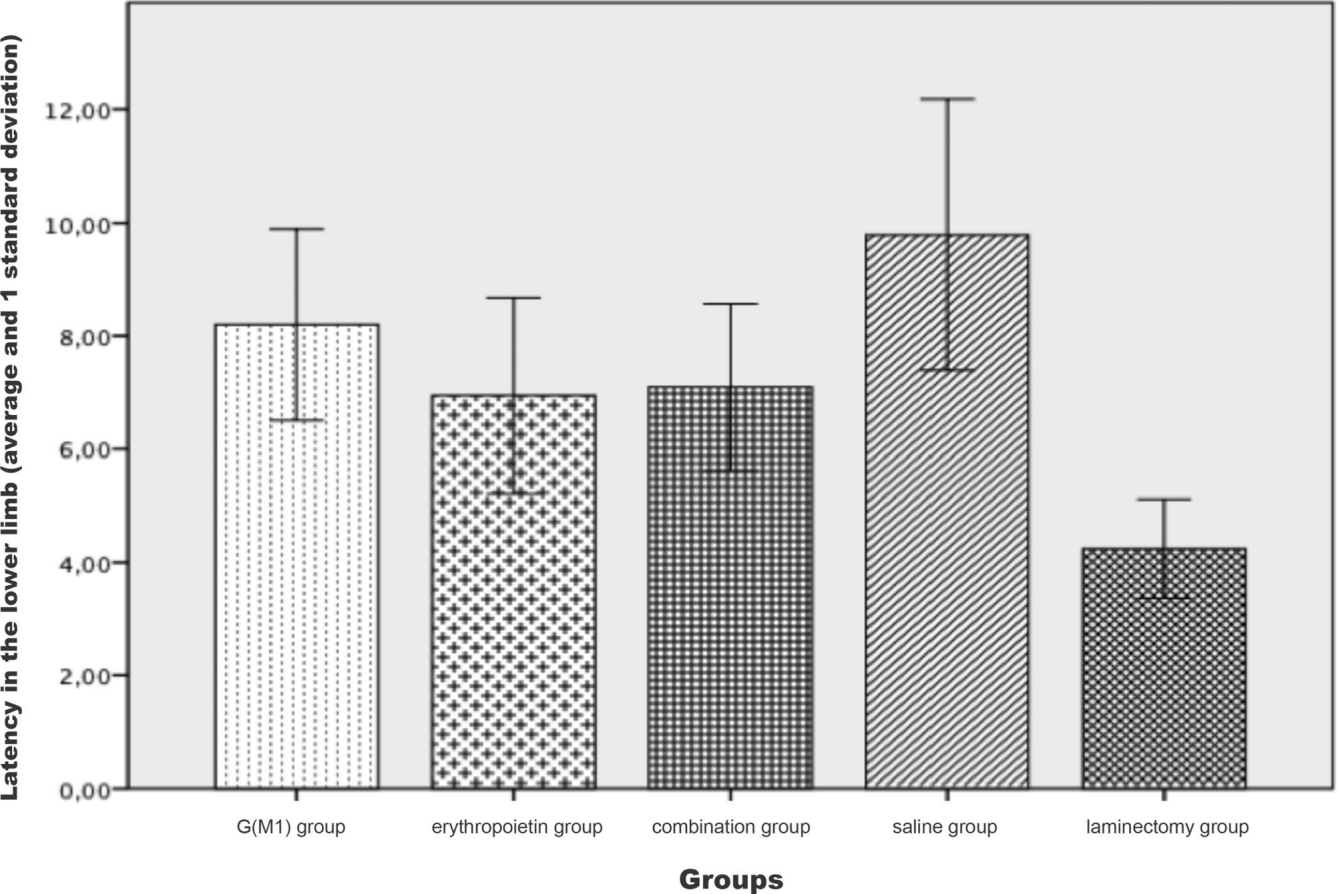
Latency, as evaluated by motor evoked potential recording, in the lower limbs of Wistar rats subjected to experimental spinal cord contusion lesioning and of control rats not undergoing experimental lesioning.

The latency of the lower limbs showed a statistically significant difference (*p*<0.01) between the groups. The Bonferroni post hoc multiple comparison test showed that the latency was higher in the saline group and was significantly lower in the laminectomy group (*p*<0.05) than in the other groups. The comparison between the three intervention groups did not show a statistically significant difference in latency (*p*>0.05), as shown in Figure 2.

**Figure 3:**
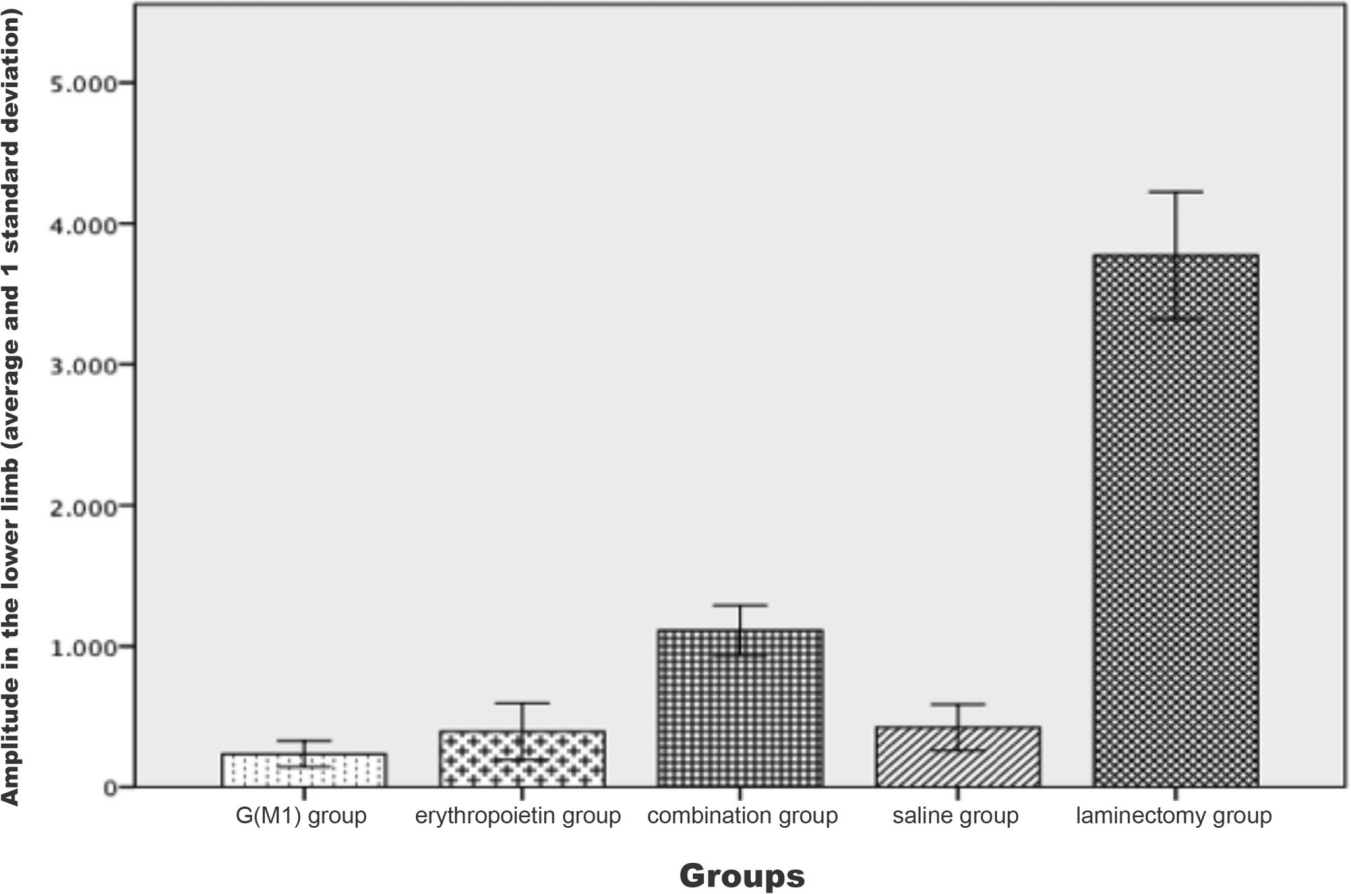
Amplitude, as evaluated by motor evoked potential recording, in the lower limbs of Wistar rats subjected to experimental spinal cord contusion lesioning and of control rats not undergoing experimental lesioning.

The signal amplitude of the lower limbs was significantly different between the groups (*p*<0.01). Based on the Bonferroni post hoc multiple comparison test, the laminectomy group showed the greatest amplitude and the saline group exhibited a lower amplitude than the three intervention groups; these differences compared to the G(M1), erythropoietin and combined groups were statistically significant (*p*<0.05). Additionally, the combined group exhibited a statistically significant difference in amplitude compared with all other groups (*p*<0.01), as shown in Figure 3.

### Histological analysis

Histological evaluation of the tissue in the area of the spinal cord lesion confirmed the presence of necrosis (Table 3), hemorrhage (Table 4), hyperemia (Table 5), degeneration of the substance (Table 6) and cellular infiltration (Table 7) in the 50 examined rats. The laminectomy group, which was not subjected to spinal cord lesioning, did not exhibit degeneration, hyperemia, hemorrhage, or necrosis. The differences in the histological scores between the groups was not statistically significant (*p*>0.05).

**Figure 4:**
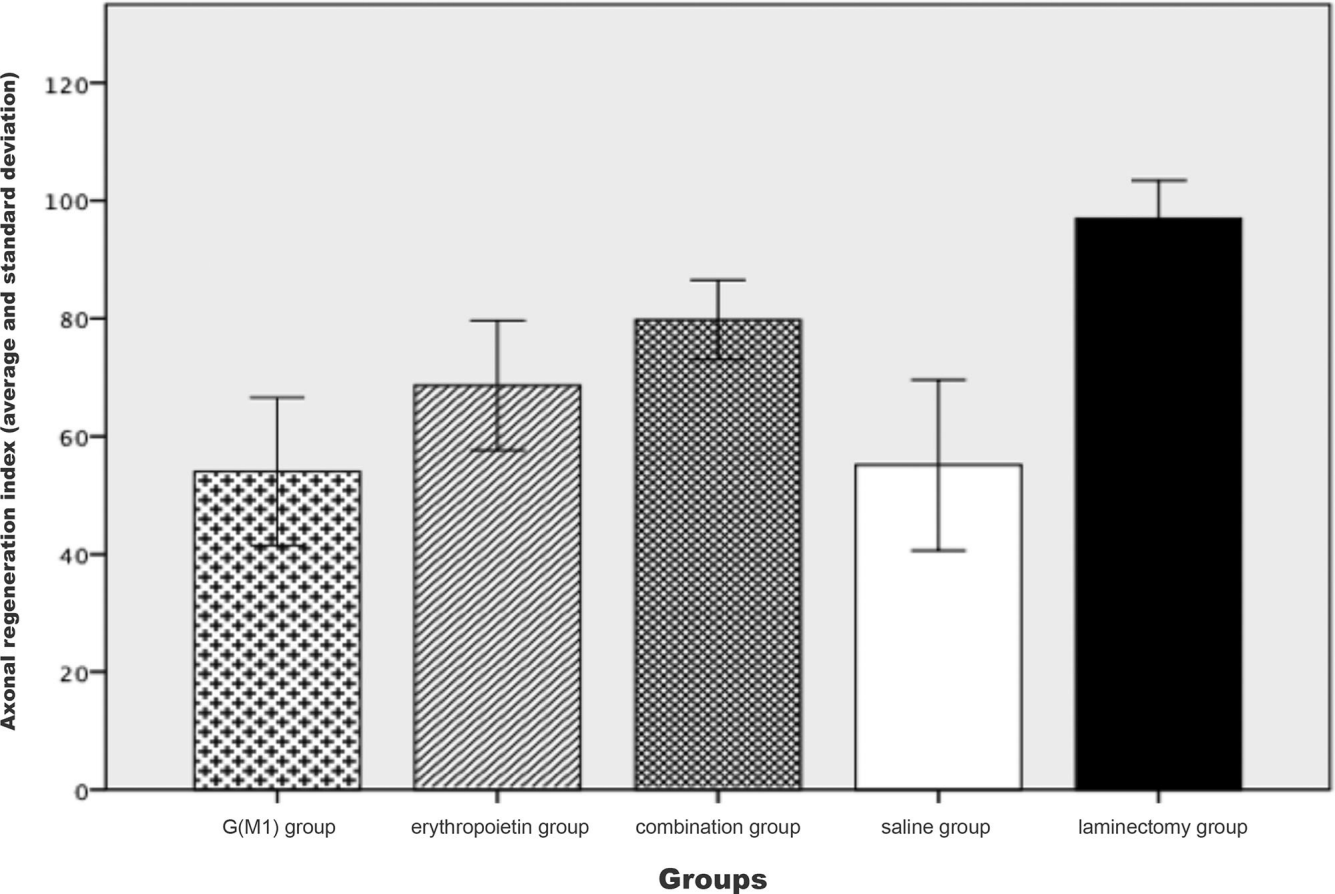
Axonal regeneration index for each group.

The Kruskal-Wallis test showed a significant difference in the axonal RI between the groups when considered together (*p*<0.01), as shown in Figure 4. As demonstrated in the figure, the saline group clearly exhibited a lower RI than the combination group. The Mann-Whitney test, which was performed for a pairwise comparison of the groups, showed that combined treatment with G(M1) and erythropoietin resulted in a significantly higher RI than any other intervention (*p*<0.01).

## DISCUSSION

In recent years, research on the topic of spinal cord lesions has shifted its focus from attempting to interrupt or delay the chain of events that results in secondary lesion formation to identifying drugs that effectively promote neuronal repair and regeneration 19. Because nervous system lesions recover slowly and incompletely 20, the current research encompasses a variety of strategies focused on reducing secondary damage and stimulating regeneration. These strategies range from physiotherapy (e.g., exercise, hypothermia and oxygen therapy), aided by the administration of compounds that may increase perfusion and stimulate angiogenic responses, to cellular therapies and forms of therapy. The outcomes of the current treatment strategies can vary depending on the species of the mammalian model used in the laboratory 10-12,14,15,21-24.

Ganglioside G(M1) and methylprednisolone comprise the only currently available compounds for the prevention of secondary spinal cord lesions in humans 25. Theoretically, in promoting regeneration, G(M1) would be considered as a “pro-inflammatory” factor, which in many cases would not be administered at the time of acute trauma. However, the goal of methylprednisolone administration is to reduce local inflammation and lipid peroxidation, opposing the activities of G(M1).

Erythropoietin, acting as a neuroprotective agent, is a component of the preventative treatment of ischemia-induced axonal death. The primary contribution of our study is the finding of the potentiating effects of combined treatment with erythropoietin and G(M1), i.e., a significant enhancement of axonal regeneration due to the combined administration of these two drugs. To our knowledge, these compounds have not yet been tested together.

In our study, beyond the first week after spinal cord lesioning, the locomotor function (measured by the BBB scale) of the animals that received erythropoietin was significantly greater than that of the rats that received G(M1). However, the group of rats that received the combined treatment exhibited greater locomotor function than all other groups subjected to spinal cord lesioning. The evidence that locomotor function continued to improve until the sixth week, especially in the group that received both G(M1) and erythropoietin, suggests that these two drugs may be effective in the long term rather than the short term.

MEPs are electrical responses of the peripheral neuromuscular pathways to stimulation of the motor cortex. MEP recording serves as an assessment of electrical conduction along the neural pathways; therefore, the results of this method can indicate a spinal cord lesion 21,26. In this test, two variables were assessed: signal latency and amplitude. Latency refers to the time required for an electrical impulse to leave the rat’s skull and reach its limbs. Rats are very small animals and the latency of any individual in a cohort is always very similar to that of the others. Additionally, any functioning nerve fiber is sufficient to carry the signal from one point to another and the impulse is recorded regardless of the number of existing fibers. For this reason, we do not consider the signal latency to be very useful in experimental studies of rats and this limitation could even lead to questioning whether to perform MEP recording in rats - except for the purpose of measuring the signal amplitude.

The signal amplitude, another parameter measured in MEP recordings, is more useful than the signal latency because the amplitude is related to the quantity of axons present that carry an electrical impulse. A spinal cord containing more axons produces a higher signal amplitude. For this reason, the groups with greater functional performance in this study also exhibited higher signal amplitudes than the groups with lower functional performance. The MEP signals are also used to confirm spinal cord lesioning in experimental studies; a decrease of 80% or more in the signal amplitude confirms the presence of a lesion 27-29. New methods are being developed to improve the accuracy of MEP recording for use specifically in rats 26,30 and to compare the effects of various anesthetics, which certainly influence the MEP recording results 30.

The signal amplitude and the BBB scores observed in this study were higher in the rats that received combined treatment with erythropoietin and G(M1). Although we demonstrated significant differences in locomotion capacity among the rats treated with the combined therapy, our results did not demonstrate a recovery at the tissue level. Histological analysis was performed on tissues stained with hematoxylin and eosin and observed under an optical microscope due to the availability of these materials. However, in both the international literature and in our laboratory experience, this technique has been shown to be deficient in terms of differentiating between specimens. The analysis of histological sections was conducted quantitatively to enable statistical analysis. However, this quantification is subjective with respect to the examined variables. We believe that if the identical analysis were performed by an additional observer or using an electron microscope, we may have obtained the same results from the histological analysis as the results of the functional tests in rats *in vivo*. However, analysis of toluidine blue-stained axons at a higher magnification showed that the rate of regeneration in our experimental model, based on the number and diameter of axons detected in the spinal cord tissue, was higher in the rats treated with both G(M1) and erythropoietin than in the rats treated with either agent alone.

The devastating nature of spinal cord lesions in humans continues to prompt thousands of studies each year and this phenomenon makes it difficult to keep track of the most promising proposals. The cascade of secondary events following a primary mechanical lesion, in addition to its initial necrotic properties, involves an apoptotic mechanism that has prompted investigations at the cellular level. These secondary events include vascular, ischemic, and homeostatic abnormalities, as well as oxidative stress and inflammatory responses. Thus, new treatment options in addition to ganglioside and methylprednisolone are needed for this injury.

We believe that there is a tendency towards increasingly multifactorial therapy for spinal cord lesions using multiple concomitant approaches and, in the case of medications, involving combined treatment with several drugs that potentiate the effects of one another. The neuroprotective effects of various compounds are currently being investigated 25. Specifically, erythropoietin and G(M1) continue to be investigated, and sodium and calcium channel blockers, minocycline, corticosteroids, progesterone, aminosteroids, opioid receptor antagonists, serotonin antagonists, thyrotropin-releasing hormone, antioxidants, free radical scavengers, growth factors, neurotrophic factors, paclitaxel, clenbuterol, gabexate mesylate, activated protein C, caspase inhibitors, tacrolimus, the copolymer polyethylene glycol (PEG) 11,25 and neurotrophins 23 have been examined. The expression of genes following spinomedullary trauma 22 and stem cell transplantation 21, which we have already begun to explore in our laboratory, may be promising paths of research.

In light of the above findings, it is clear to us that a “cure” for spinomedullary trauma will be complex and unfortunately will not be accomplished by a single form of therapy. In addition to pharmacological treatment, physical and biological therapies, or even the use of robotics as a component of treatment, should be included the treatment approach. Unfortunately and contrary to the very optimistic forecasts that expected a cure for spinomedullary trauma by the end of the “Decade of the Spine” (2001 to 2010), it has become increasingly clear that a definitive solution for this problem remains a long way off. Collaborative, multidisciplinary studies, such as the one presented in this report, form the building blocks that will help to develop a definitive “cure” for spinomedullary trauma.

This study showed that G(M1) and erythropoietin exert therapeutic effects on motor and electrophysiological function and on axonal regeneration in Wistar rats subjected to experimental spinal cord lesioning. Moreover, combined treatment with these two agents potentiates their effects.

## AUTHOR CONTRIBUTIONS

Marcon RM designed the study, collected data, interpreted the results, wrote the manuscript and approved the final version to be published. Cristante AF contributed to the study design and data interpretation, helped with manuscript writing and approved the final version to be published. Barros Filho TE contributed to data interpretation, manuscript writing and critical revision and approved the final version to be published. Ferreira R and Santos GB collected data, interpreted the results, revised the manuscript and approved the final version to be published. The manuscript was produced, reviewed and approved by all the authors collectively.

## Figures and Tables

**Table 1:** Motor evoked potential results: latency in the lower limbs in each group.

	Mean	SD	SE	95% CI		Minimum	Maximum
				IL	SL		
G(M1)	8.2000	1.69395	0.37878	7.4072	8.9928	4.50	12.50
ERI	6.9400	1.73278	0.38746	6.1290	7.7510	3.50	10.00
COMB	7.0900	1.47609	0.33006	6.3992	7.7808	4.10	10.20
SS	9.7850	2.39611	0.53579	8.6636	10.9064	6.30	16.30
LAM	4.2400	0.87503	0.19566	3.8305	4.6495	2.60	6.10
**Total**	7.2510	2.47576	0.24758	6.7598	7.7422	2.60	16.30

G(M1) = ganglioside G(M1); ERI = erythropoietin; COMB = combination of ganglioside G(M1) and erythropoietin; SS = saline solution; LAM = laminectomy; 95% CI = 95% confidence interval; SD = standard deviation; SE = standard error; IL = inferior limit; SL = superior limit.

**Table 2:** Motor evoked potential results: amplitude in the lower limbs in each group.

	Mean	SD	SE	95% CI		Minimum	Maximum
				IL	SL		
G(M1)	235.2500	92.03082	20.57872	192.1783	278.3217	122.00	425.00
ERI	393.4000	201.76312	45.11561	298.9720	487.8280	215.00	854.00
COMB	1110.5500	178.84644	39.99128	1026.8473	1194.2527	785.00	1452.00
SS	422.8000	164.70247	36.82859	345.7169	499.8831	152.00	699.00
LAM	3772.8500	452.91015	101.27379	3560.8815	3984.8185	2895.00	4521.00
**Total**	1186.9700	1356.60324	135.66032	917.7905	1456.1495	122.00	4521.00

G(M1) = ganglioside G(M1); ERI = erythropoietin; COMB = combination of ganglioside G(M1) and erythropoietin; SS = saline solution; LAM = laminectomy; 95% CI = 95% confidence interval; SD = standard deviation; SE = standard error; IL = inferior limit; SL = superior limit.

**Table 3:** Necrosis in the distal section of the spinal cord in Wistar rats subjected to experimental spinal cord contusion lesioning.

Group							
Necrosis		G(M1)	ERI	Combined	Saline	Laminectomy	Total
Absent	Count	2	3	4	2	10	21
	% Necrosis	9.5%	14.3%	19.0%	9.5%	47.6%	100.0%
	% Group	20.0%	30.0%	40.0%	20.0%	100.0%	42.0%
Mild	Count	2	4	4	3	0	13
	% Necrosis	15.4%	30.8%	30.8%	23.1%	0.0%	100.0%
	% Group	20.0%	40.0%	40.0%	30.0%	0.0%	26.0%
Moderate	Count	5	2	2	3	0	12
	% Necrosis	41.7%	16.7%	16.7%	25.0%	0.0%	100.0%
	% Group	50.0%	20.0%	20.0%	30.0%	0.0%	24.0%
Intense	Count	1	1	0	2	0	4
	% Necrosis	25.0%	25.0%	0.0%	50.0%	0.0%	100.0%
	% Group	10.0%	10.0%	0.0%	20.0%	0.0%	8.0%
Total	Count	10	10	10	10	10	50
	% Necrosis	20.0%	20.0%	20.0%	20.0%	20.0%	100.0%
	% Group	100.0%	100.0%	100.0%	100.0%	100.0%	100.0%

G(M1) = ganglioside G(M1); ERI = erythropoietin.

**Table 4:** Hemorrhage in the distal section of the spinal cord in Wistar rats subjected to experimental spinal cord contusion lesioning.

Group							
Hemorrhage		G(M1)	ERI	Combined	Saline	Laminectomy	Total
Absent	Count	1	4	4	3	10	22
	% Necrosis	4.5%	18.2%	18.2%	13.6%	45.5%	100.0%
	% Group	11.1%	40.0%	40.0%	30.0%	100.0%	44.9%
Mild	Count	3	3	4	4	0	14
	% Necrosis	21.4%	21.4%	28.6%	28.6%	0.0%	100.0%
	% Group	33.3%	30.0%	40.0%	40.0%	0.0%	28.6%
Moderate	Count	2	3	1	2	0	8
	% Necrosis	25.0%	37.5%	12.5%	25.0%	0.0%	100.0%
	% Group	22.2%	30.0%	10.0%	20.0%	0.0%	16.3%
Intense	Count	3	0	1	1	0	5
	% Necrosis	60.0%	0.0%	20.0%	20.0%	0.0%	100.0%
	% Group	33.3%	0.0%	10.0%	10.0%	0.0%	10.2%
Total	Count	9	10	10	10	10	49
	% Necrosis	18.4%	20.4%	20.4%	20.4%	20.4%	100.0%
	% Group	100.0%	100.0%	100.0%	100.0%	100.0%	100.0%

G(M1) = ganglioside G(M1); ERI = erythropoietin.

**Table 5:** Hyperemia in the distal section of the spinal cord in Wistar rats subjected to experimental spinal cord contusion lesioning.

Group							
Hyperemia		G(M1)	ERI	Combined	Saline	Laminectomy	Total
Absent	Count	2	4	5	2	10	23
	% Necrosis	8.7%	17.4%	21.7%	8.7%	43.5%	100.0%
	% Group	20.0%	40.0%	50.0%	20.0%	100.0%	46.0%
Mild	Count	4	5	4	4	0	17
	% Necrosis	23.5%	29.4%	23.5%	23.5%	0.0%	100.0%
	% Group	40.0%	50.0%	40.0%	40.0%	0.0%	34.0%
Moderate	Count	1	1	1	3	0	5
	% Necrosis	20.0%	20.0%	20.0%	40.0%	0.0%	100.0%
	% Group	10.0%	10.0%	10.0%	20.0%	0.0%	10.0%
Intense	Count	3	0	0	2	0	5
	% Necrosis	60.0%	0.0%	0.0%	40.0%	0.0%	100.0%
	% Group	30.0%	0.0%	0.0%	20.0%	0.0%	10.0%
Total	Count	10	10	10	10	10	50
	% Necrosis	20.0%	20.0%	20.0%	20.0%	20.0%	100.0%
	% Group	100.0%	100.0%	100.0%	100.0%	100.0%	100.0%

G(M1) = ganglioside G(M1); ERI = erythropoietin.

**Table 6:** Axonal degeneration in the distal section of the spinal cord in Wistar rats subjected to experimental spinal cord contusion lesioning.

Group							
Degeneration		G(M1)	ERI	Combined	Saline	Laminectomy	Total
Absent	Count	2	3	3	0	10	18
	% Necrosis	11.1%	16.7%	16.7%	0.0%	55.6%	100.0%
	% Group	20.0%	30.0%	30.0%	0.0%	100.0%	36.0%
Mild	Count	4	6	6	6	0	22
	% Necrosis	18.2%	27.3%	27.3%	27.3%	0.0%	100.0%
	% Group	40.0%	60.0%	60.0%	60.0%	0.0%	44.0%
Moderate	Count	3	1	1	3	0	8
	% Necrosis	37.5%	12.5%	12.5%	37.5%	0.0%	100.0%
	% Group	30.0%	10.0%	10.0%	30.0%	0.0%	16.0%
Intense	Count	1	0	0	1	0	2
	% Necrosis	50.0%	0.0%	0.0%	50.0%	0.0%	100.0%
	% Group	10.0%	0.0%	0.0%	10.0%	0.0%	4.0%
Total	Count	10	10	10	10	10	50
	% Necrosis	20.0%	20.0%	20.0%	20.0%	20.0%	100.0%
	% Group	100.0%	100.0%	100.0%	100.0%	100.0%	100.0%

G(M1) = ganglioside G(M1); ERI = erythropoietin.

**Table 7:** Cellular infiltration into the distal section of the spinal cord in Wistar rats subjected to experimental spinal cord contusion lesioning.

Group							
Infiltration		G(M1)	ERI	Combined	Saline	Laminectomy	Total
Absent	Count	4	5	6	2	8	25
	% Necrosis	16.0%	20.0%	24.0%	8.0%	32.0%	100.0%
	% Group	40.0%	50.0%	60.0%	20.0%	80.0%	50.0%
Mild	Count	3	3	4	5	2	17
	% Necrosis	17.6%	17.6%	23.5%	29.4%	11.8%	100.0%
	% Group	30.0%	30.0%	40.0%	50.0%	20.0%	34.0%
Moderate	Count	3	2	0	3	0	8
	% Necrosis	37.5%	25.0%	0.0%	37.5%	0.0%	100.0%
	% Group	30.0%	20.0%	0.0%	30.0%	0.0%	16.0%
Intense	Count	10	10	10	10	10	50
	% Necrosis	20.0%	20.0%	20.0%	20.0%	20.0%	100.0%
	% Group	100.0%	100.0%	100.0%	100.0%	100.0%	100.0%

G(M1) = ganglioside G(M1); ERI = erythropoietin.
